# Medical Society of the County of Erie

**Published:** 1891-07

**Authors:** B. H. Grove

**Affiliations:** Secretary pro tem.


### MEDICAL SOCIETY OF THE COUNTY OF ERIE.

*Special Meeting, held at Y. M. C. A. Hall, May 16, 1891.*

Reported by B. H. GROVE, M. D., Secretary *pro tem.*

The President, Dr. E. C. W. O'Brien, called the meeting to order, and Dr. B. H. Grove was appointed Secretary *pro tem.*

The following-named physicians were present: Drs. Cronyn, Hopkins, Hayd, Howe, Dorland, Grosvenor, J. C. Greene, S. S. Greene, Abbott, Cary, Harvey, O'Brien, Parmenter, Howe, Pattison, Rochester, Stockton, Snow, Phelps, Coakley, Goldberg, Falk, Camehl, Hubbell, J. S. Smith, E. A. Smith, Bissell, York, Crawford, Fowler, Lothrop, W. W. Potter, Thornton, Johnson, Mulford, Lapp, McPherson, Walsh, L. P. Dayton, Bartlett, Mackey, Mynter, and Wright.

The President explained the purpose of the meeting, as follows:

### REMARKS OF DR. E. C. W. O'BRIEN.

*Gentlemen*—You are all aware of the sad cause of our gathering here to-night, for the sorrowful tidings of Dr. Burwell's death last evening was quickly known in all portions of our city. It was melancholy news to all who knew him, but to none more so than to his medical brethren, for it is known to us all how greatly we respected him, and how well we loved him. I know that there are many here, especially among the honored elders of our profession, who will speak of him to-night as they knew him, and that all their words will be an eloquent endeavor to do justice to his memory; yet, I feel that I, also, could not be content unless I give my hearty tribute to his great personal worth. Dr. Burwell was a delightful character, making the most pleasant impression upon all with whom he came in contact. He was esteemed to an unusual degree by the people of this city, where he was in active and successful practice for almost fifty years, and he was loved by his patients to a degree not often vouchsafed even to the most faithful and fortunate practitioner. His patients could not help loving him, for he had special traits of character which readily won their enduring affection. He was in various ways an ideal physician, though his practice was so very large, (much larger than that of most physicians, even skilful and noted ones,) and though he conducted that practice in the most quiet and unobtrusive manner, still we knew he was a constant student, even to the last weeks of his useful life, and he was abreast of modern thoughts and progress in his profession to a degree that is unusual for a man of his advanced years and busy career. And he set a noble example to his professional brethren, in fact, to all men, in the purity of his daily life. And we all know the inestimable value of such an example, especially to the younger members of our profession, as well as upon his fellow-citizens, of such a man as Dr. Burwell. His skill, his genial presence, his words of encouragement, will be missed from many a sick-room and from many a home where he has been the trusted physician, counselor and friend. He was as well known in the humble homes of the poor as in the mansions of the rich. The poor especially will never forget him. His deportment was an example. The physician, like the clergyman, comes into confidential relations in our homes ; like the clergyman, he should come there with clean hands and heart. Dr. Burwell was known to us as a virtuous, christian gentleman; his mind was pure, and I believe that every word he ever uttered was fit to be spoken in a temple of our Lord.

Dr. James W. Putnam moved that a committee of three be appointed by the Chair to draft resolutions expressive of the sense of the Society in regard to the sad event that convenes it.

Carried ; and the President appointed as such committee Drs. John Cronyn, F. W. Bartlett, and John Hauenstein.

Dr. Henry Lapp, of Clarence, reported the death of Dr. A. F. Helwig, of Swormville, and moved that a committee be appointed to report resolutions, and also one to attend the funeral.

Drs. Henry Lapp, S. S. Greene, J. B. Coakley, E. T. Dorland, and Callanan were appointed to draft resolutions, and Drs. Lapp, Martin, and Myers, of Clarence ; Drs. McPherson and Simon, of Akron ; Drs. Bowman and Tyler, of Alden ; Drs. Trull and Carmer, of Williamsville ; and Drs. Parker and Krauss, of the Erie County Almshouse, as a committee to attend the funeral.

The first appointed committee reported the following :

### MEMORIAL OF DR. GEORGE N. BURWELL.

Whereas, In the inscrutable dispensation of Providence it is decreed that our universally esteemed brother practitioner, Dr. George

N. Burwell, has been removed from the toil and labor of his daily life, with which he was so engaged in his great desire to remove or ameliorate the ills of his suffering patients ; that, like his efforts to alleviate human suffering, he himself passed away in the quietness peculiar to his placid nature. Therefore,

*Resolved,* That the knowledge we here have of his life be as a beacon to guide us in emulation thereof, and that our deep grief at his loss be represented as our condolence to his friends and relatives.

*Resolved,* That this Society attend his funeral in a body, as a testimony of his worth and its esteem.

(Signed,) JOHN CRONYN,
F. W. BARTLETT,
JOHN HAUENSTEIN.

Dr. J. C. Greene was glad to bear testimony to the worth of Dr. Burwell. He (Dr. Greene) came to the city when a young man, and soon thereafter met Dr. Burwell in consultation. He called to mind his many words of cheer and consolation. He was told there was plenty of room in the city for one more, and that he should try and climb to the top round. Dr. Greene always found Dr. Burwell a safe adviser, and he was never known to obtain patients by unfair means. He located in a district of the city where Dr. Burwell did faithful work. He was honest, but not ambitious.

Dr. James W. Putnam: The position in which I am placed is a peculiar one. I looked upon Dr. Burwell as a professional father, as a counselor, and I experience in his death a personal loss. I have known him for eleven years. As I learned to know him better, I learned to reverence him more and to appreciate his worth. He did many acts of kindness and charity in a humble way. His gifts to the poor and sick he called completing the cure. He was especially kind to the young men of the profession. He had but little personal ambition, and was content to do his day's work faithfully and well. He was in touch with the modern investigations, and a constant reader of the medical journals.

Dr. H. E. Hayd: We have listened with approbation to the many eulogies of Dr. Burwell. I knew none of the older men of our profession better, or loved them more. Whenever I met him my esteem became greater. It was pleasant to consult with him, for there was never an insinuation that anything was wrong. His powers of perception and observation were good. His ripe judgment always did the physician and patient good. I have often come in close contact with him and was impressed with his lovely disposition.

To us younger men he is a fitting example. He was true to every professional obligation, and to humanity in general.

Dr. Lucien Howe: There is but little new that we can say of each other. We help some to get better or worse. A professional brother passes away, and we fill up the ranks. Dr. Burwell served as a landmark. We, who have been here seventeen years and more, realize that many have gone from us. We call to mind the honorable contests of Drs. Miner, Rochester, Loomis, and Burwell. When such a man as Dr. Burwell dies, a guide-post is taken away. He had grown lusty and strong in conflict. It is noteworthy that, during a professional life of fifty years, there is cause for so much of eulogy and so little of detriment. His life abounded in acts of kindness and love, which endeared him both to members of the profession and his patients.

Dr. Rochester: I learned to know Dr. Burwell intimately when he was taking care of members of my family. I have experienced his love, and in the relationship of patient to physician I want to pay my tribute of respect to his memory.

Dr. S. S. Greene: It was often my privilege to call Dr. Burwell in consultation. He seemed like a father to me and his patients. He was held in much esteem, and the large number of us assembled this evening is an evidence of the affection entertained for him.

Dr. E. T. Dorland: I wish to add my mite in eulogy of Dr. Burwell. My acquaintance with him dates back to my boyhood. I knew him while living at Hamburg. I can testify personally to his many acts of kindness. In fact, I owe my life to his extreme care when sick with typhoid fever. He was always solicitous for my welfare, and, every other day, would drive out to my home to visit me.

Dr. H. R. Hopkins: It seems important that members should be free from exaggerations on such occasions as the present. I trust, therefore, the members of this Society will bear me out in my effort to speak with exactness. In the year 1860, there were giants in the profession. The training of men was different then than now. The pioneers also bring a result different from us who are pampered with all the modern conveniences of travel. There was then conflict and contests, and from these there have grown principles. I came to Buffalo early in the 'sixties, and at that time no man was more conspicuous than Dr. Burwell; no man was more rugged or of better professional character. We associate character with certain lineaments, and the face of our departed brother was expressive of love and sympathy. Dr. Burwell manifested remarkable industry, and while attending physician at the hospital would frequently visit the institution before breakfast. He was devoted to his profession, and was incorruptible. His life was an example of modesty and manliness, and was replete with professional acts of kindness.

Dr. A. R. Wright: I followed Dr. Burwell's clinic years ago, and found him a careful man in his observations. There was nothing done in a careless manner, and his opinions were good and reliable. Dr. Burwell had just returned from Europe, and was considered an adept. As a pathologist, he was correct and able. Although not a member of this Society, I am pleased to bear this slight tribute to the memory of our departed friend, and thank you for this opportunity.

Dr. F. W. Bartlett: I lived ten years next door to Dr. Burwell. I can bear testimony to his noble character. He possessed an unusual amount of industry and the happy faculty of attaching himself to his patients. He was faithful to the last degree, and there was a depth of affection in his heart. He was amiable in character, and free from vain ambition. He was faithful, day in and day out, and worthy of great emulation. Let us cherish his memory.

Dr. Herman Mynter: We all knew Dr. Burwell to love him, and we trusted him implicitly. In typhoid fever or pneumonia, we would have called him in as our adviser. He was a man of the best repute, and a type for honorable professional conduct.

Dr. John Cronyn: I speak upon this subject of Dr. Burwell's death with feelings full of grief. We were the warmest of friends. No one knew him better than myself, and I always found him a thorough gentleman. The men I knew forty years ago are dropping away. Drs. Flint, White, Rochester, Loomis, and now Dr. Burwell, have passed away. They were giants in the profession. We used to hear lucid discussions from these gentlemen on medical subjects; and now, of all the men that used to meet for conference on South Division street years ago, I am the only one left, excepting, possibly, Dr. John Hauenstein.

Dr. Charles Cary: Dr. Burwell was known thoroughly to the profession. I have known him from childhood, and our relationship has been intimate. He was a keen observer and a hard student. When a boy my father assured me that the life of Dr. Burwell was worthy of emulation. I have watched him, and I have seen the truth demonstrated. He never treasured ill-feeling against any one; neither did there exist in his soul any ill-will against the Buffalo Medical University. He was, in fact, the first charter subscriber to the college fund.

Dr. J. B. Coakley: Our departed brother practitioner possessed a splendid character, and his bearing towards the profession was noble. I learned to know him ten years ago when I came to the city. He called upon me and gave evidence of his patriotism and largeness of heart. He possessed ability as a leader, but was inclined to be quiet and unassuming.

Dr. W. T. Callanan: In referring to the death of Dr. Burwell and the eulogies which have been expressed this evening, it has occurred to me that a record of the lives of the most prominent members of this Society should be preserved among the archives of this Society.

Dr. S. W. Wetmore sent a letter of regret at his unavoidable absence, in which he also paid tribute to the memory of Dr. Burwell.

The second committee reported the following:

### MEMORIAL OF DR. A. F. HELWIG.

This Society having heard of the all too early death of Dr. A. F. Helwig, of Swormville, records its profound sorrow in learning of the decease of this very promising member. He was an industrious student, a faithful practitioner, ever ready to attend the poor. He was a true Christian, and this Association deeply deplores his loss.

We extend to his wife and family our profound sympathy in this sad hour.

(Signed,) HENRY LAPP. S. S. GREENE.
J. B. COAKLEY. E. T. DORLAND.
W. C. CALLANAN.

On motion of Dr. Cary, the Society then adjourned.

---

### STATED MEETING.

Reported by ELI H. LONG, M. D, Secretary.

Regular semi-annual meeting held at the lecture hall of the Y. M. C. A., West Mohawk Street, June 9, 1891.

The Vice-President, Dr. W. H. Gail, of East Aurora, in the chair.

The minutes of the last annual meeting were read, and after debate in an unsuccessful attempt to amend, were adopted as read.

The minutes of the special meetings, held March 20th and May 16th, were adopted.

The Committee on Membership recommended the admission of the following-named physicians: R. L. Patteson, Walter J. Riehl, Earl G. Danser, William Dowlman, H. S. Townsend, Wm. P. Clothier, Jno. J. McCullough, John Honsberger, Edson H. Young, Wm. H. Chace, C. R. Jennings, J. F. Sell, M. J. O'Connell, J. P. Wilson, and Wm. H. Woodbury. Upon motion they were elected to membership and those present were introduced to the Society. Upon motion of Dr. Hubbell an omission in the minutes of the last annual meeting was corrected by the insertion of the name of Dr. Edwin A. Millring in the list of those elected at that meeting.

Upon motion of Dr. Storck, the official list of members was ordered corrected by the insertion of the name of Dr. Chas. H. W. Auel, who had been regularly elected at a previous meeting.

The following-named physicians presented applications in regular form, which were referred to the committee on membership:

Harriott E. Sheldon, Fred Hamilton Powell, Charles A. Meahl, E. H. Tweedy, Charles H. Woodard, Charles P. Clark, Frank E. Hill, Lillian C. Randall, Lewis G. Smith, Andrew B. Kinsley, Irving W. Potter, Frederick Preiss, Charles J. Reynolds, John R. Gray, Arthur B. Allen, Francis L. Watkins, Charles A. Schladermundt.

### REPORT OF THE BOARD OF CENSORS.

At the last annual meeting of this Society, your Board of Censors stated in its report that some of our members were supposed to be connected with certain irregular so-called medical institutes in this city, in violation of the by-laws and medical ethics of the Society, and, if sufficient proof could be obtained, charges would be prepared against them at this meeting.

The Board of Censors at its last meeting directed its chairman to prefer charges against Dr. Isaac G. Wheeler, 191 E. Eagle street, and Dr. Bernard Cohen, 336 Spring street, both members of this Society, for the violation of Sections 3, 5 and 8, of Article VII., of the By-laws of this Society, and accordingly we present the following specifications:

1. Dr. Isaac G. Wheeler has been for some time past associated in practice and in business with two irregular and illegal practitioners, named Horace Dewey and Ellen Crossley, who advertised and managed a "Catarrh and Dyspepsia Institute," at 133 E. Eagle, cor. Elm street, Buffalo, N. Y.; that he occupied the same office with them, prescribed and prepared medicines for their patients, and received money for giving protection by his diploma to this unlawful firm. When the Board of Censors notified them that they were practising in violation of the State medical law, and would be prosecuted, we were informed through their legal adviser that "their institute was all right, as it was managed *by a regular graduate, a member of this Society*." This was rather a surprise for your Board, but we were determined to find out who *our member*, the *protector* of this apparently illegal concern, was. Through the services of an efficient detective of our police department, conclusive evidence and proof was furnished that it was Dr. Isaac G. Wheeler. His associates were Horace Dewey and Mrs. Ellen Crossley, who have been since indicted by the grand jury for violating the State medical law.

2. Dr. BERNARD COHEN has been, for some time past, and is now, associated in practice and in business with an irregular practitioner, named Ferdinand Simon, 336 Spring street, who has been indicted by the grand jury for violation of the State medical law, was arraigned before the court and plead guilty of the indictment. It was ascertained that Dr. Cohen practised with Simon in the same office, and received money of him for protecting the firm with his diploma. They have now an advertisement in a German paper published in East Buffalo, of which the following is a translation: "*The Simon Medical Company, Water-Doctors*, 336 Spring street. ☞ Bring your water along, and we will find out what your sickness is."

Your Board of Censors has notified the members accused that such charges and specifications would be preferred against them at this meeting; that they might appear before the Society to make answer and defense in their behalf.

The Board would further report that since the January meeting, five complaints against persons practising in this county in violation of the State medical law have been filed in the District Attorney's office, with full proofs and evidence of the offense for prosecution. Previous to the 27th of May last, only one case, that of Ferdinand Simon, was brought before court for trial, and, although he plead guilty to the indictment, he was not sentenced by the judge. With this result your Board could have made only a very unsatisfactory report at this meeting. The chairman, therefore, appealed to District Attorney George T. Quinby for a written statement in regard to the treatment of the other cases in his office. In answer the Board was furnished by Mr. Quinby with the following report hereto attached, which will give the Society the necessary information:

OFFICE OF THE DISTRICT ATTORNEY,
Buffalo, N. Y., May 29, 1891.

Dr. E. STORCK, Buffalo, N. Y.:

DEAR SIR—In answer to your letter of the 27th inst., in regard to cases in this office, I desire to say that all five cases presented by you for prosecution at this office were fully and fairly presented to the grand jury for their consideration. Three were indicted, and for the failure to indict the others this office cannot be held responsible; that responsibility must rest upon the grand jury. This office pressed for trial the case against Ferdinand Simon, and he plead guilty; but Judge Seaver, in the exercise of his discretion, allowed him to go upon suspended sentence. This office is not responsible for the exercise of judicial discretion on the part of Judge Seaver. Mr. Kenefick informs me that you did notify Judge Seaver that Simon was practising again, and that the Judge promised to have him brought up before him at the May Term. The first two weeks of the May Term are taken up with civil business. I have no doubt that the Simon case will be disposed of when the criminal business is taken up next Monday, June 8th. Horace Dewey was indicted, a bench warrant was issued for his arrest, but the Sheriff has been unable to find him, and the warrant is still in his hands. If you can give us or the Sheriff any information as to his whereabouts, we will have him arrested and prosecuted. In the case of Ellen Crossley, it was some time after the grand jury indicted her before she was found. Her case was moved for trial at the last Term of the Superior Court. Her attorney moved for a commission to take the testimony of a witness in Pittsburg. Judge Hatch granted the motion and a commission was issued. This necessitated a postponement of the trial, which we shall move next week if the testimony is returned.

It will thus be seen that there has been no negligence on the part of this office, and no desire to delay the prosecution of these cases.

Yours respectfully,
GEO. T. QUINBY.

One of the cases the District Attorney refers to, where the grand jury has failed to indict, and in which he places the responsibility where it belongs, is that of the Clairvoyant Doctress, Mrs. Matteson, on North Division street. Conclusive evidence and proof was presented of her guilt; it was shown that she had practised in violation of the law without license or diploma, but because one of the twenty-four men—true and conscientious—claimed that she had once cured him of rheumatism, the District Attorney failed in securing twelve votes, the requisite number of this intelligent body of men to indict! The Board also has informed Dr. Francisco Palmiere, an Italian practitioner of this city, that he was practising in violation of the law, and was subject to prosecution, as he had not registered in the County Clerk's Office. The

Board questioned the legality of his diploma that he presented at the time.

The President of the Medical Faculty of Niagara University refused to endorse it more than a year ago, for the same reason, and although the diploma is now endorsed by Dr. Charles Cary, of the Buffalo Medical College, Dr. Palmiere never had it registered. The diploma is now before the State Board of Regents, where it will receive due attention and examination.

Charges have again been made to the Board against Rud. Waldvogel, a medical student in the office of Dr. Charles Weil, for practising on his own account in violation of the law. The accused has already been before Police Court on the same complaint, and sentence was suspended on his promise to desist from practising until he had graduated. Charges will be preferred against him by this Board to the District Attorney, unless he complies with the law.

Respectfully submitted,

(Signed,) E. STORCK,
ROLLIN L. BANTA,
HENRY LAPP,
M. B. FOLWELL,
EDWARD H. BALLOU.
*The Board of Censors.*

A complaint against Dr. J. M. Falk was referred to the Board of Censors for investigation.

On motion of the Chairman of the Board of Censors, seconded by Dr. Callanan, the Society proceeded to the trial of Dr. Cohen upon charges contained in the report. At this point a stranger arose and asked the privilege of the floor, to which objection was made, and the request was denied.

Dr. COHEN, being called upon to answer, stated that he took Simon's office after the latter had been ordered by Judge Seaver to discontinue practice, and that he found it necessary, in order to hold the business of his predecessor, to use his name. He, therefore, practised under the name — The F. R. Simon Medical Co., Dr. B. Cohen, attendant. He stated that according to his knowledge Mr. Simon had not been in practice since he was ordered to stop, but at present was under his preceptorship. Dr. Cohen admitted that he had placed the advertisement in the paper and was sorry for it. Upon being questioned, he stated that in the future he would practise under his own name and not advertise. In course of his defense he stated that in his relation to Simon, he had done no more than some other members who had visited patients for Simon.

He mentioned the name of one member present, who promptly denied ever having visited a patient for Simon.

A motion to expel having been offered by the Chairman of the Board of Censors, Dr. Fell advised leniency in view of this being a first offence, and he offered an amendment, substituting censure for expulsion; but a point raised by Dr. Storck that the vote must be either for expulsion or acquittal, was sustained by the Chair.

Dr. ABBOTT here raised the point whether a member in arrears was entitled to introduce a resolution. The chair ruled that he was not. A ballot was then ordered, Drs. Phelps and Fell acting as tellers.

The ballot resulted as follows: For expulsion, 51; for acquittal, 12; blank, 1. Dr. Cohen was declared expelled from the Society.

While the above vote was being counted, Dr. Crego asked unanimous consent to introduce a matter from the committee appointed to arrange entertainment for the members of the Central New York Medical Association. He stated that the committee had solicited subscriptions to meet the expenses of entertainment, but there still remained a deficit of nearly $20. It was moved and carried that the Treasurer be authorized to pay an amount not to exceed $20 to meet the deficit.

Dr. HOWE asked the privilege of placing a patient upon exhibition, which was granted. Upon motion of Dr. Callanan, seconded by Dr. Crego, it was voted that at the next annual meeting, two sessions be held, and the appointment of a committee of five to arrange a literary programme was authorized. The Chair appointed as such committee: Drs. F. S. Crego, W. C. Callanan, DeLancey Rochester, Edward Clark, and A. E. Persons.

The question was asked whether a member can resign from the Society.

The Chair called upon Dr. Storck to answer, who, with full reference to the by-laws, stated that a member could not resign.

Dr. STORCK moved that when the Society adjourns it be to meet at 3.30 P.M. Carried.

The Society then adjourned.

### AFTERNOON SESSION.

A quorum being assembled at 3.45 P.M., the meeting was called to order by Vice-President Gail.

In continuance of the consideration of matters presented in the Censors' report, the Chairman of the Board moved that Dr. Isaac

G. Wheeler be expelled from the Society; seconded by Dr. Ira C. Brown.

Dr. WHEELER having been invited to present his defense, stated that he desired to face the Society and state the facts in truth and candor. Something more than a year ago a man named Dewey called to ask him to take charge of an establishment, wishing to employ his name and services. He refused to accept the offer. At a second visit Dewey asked what arrangements he would consent to, when he told him that if he could be unrestricted in the care of patients he would consent to take charge, but that he would neither advise nor associate with him professionally, nor would he, under any circumstances, be a party to fraud or imposition of any kind.

The necessity of making this defense was a humiliation to him, but he felt that his course was to abide by the decision of the Society.

Several questions asked Dr. Wheeler brought out the facts that he had been employed on a regular salary, receiving no fees, and that he held no consultations with Dewey; further, that his connection with the concern had entirely ceased.

A ballot was ordered, Drs. Dorland and C. A. Ring acting as tellers. Drs. A. Dagenais, C. E. Ernest, R. L. Patteson, A. L. Benedict and H. D. Ingraham, by vote of the Society, were excused from voting, as they arrived too late to learn the facts in the case. The result was as follows: For acquittal, twenty-two; for expulsion, five; blank, one. Motion declared lost.

While pending the counting of the vote, communications were presented as follows:

From the Secretary of the Medical Society of the State of New York, containing announcements in reference to the Transactions of the Society for 1891, offering back volumes of the Transactions which may be needed to complete the file of the County Society, and announcing chairman of Prize-Essay Committee, with conditions of competition. The communication was received.

A letter from the Secretary of the Eighth District Dental Society, enclosing the following preamble and resolution adopted by that body:

*Whereas*, Having observed that certain men who practise dentistry by the methods of quackery may secure recommendation for professional proficiency, for publication over the names of well-known members of the medical profession;

*Therefore*, We, of the dental profession, members of the Eighth

District Dental Society of the State of New York, in regular convention assembled, have

RESOLVED, That, in our opinion, it is not in accordance with the usually accepted code of ethics for any professional man to furnish, for publication, certificates extolling or vouching for the professional qualifications of any other professional man, thereby perhaps bringing upon others a professional scandal.

We desire most respectfully to call the attention of physicians to this matter.

The communication was received and placed on file.

The resignation of Dr. Cohen from membership was read, and laid on the table. Under Miscellaneous Business it was moved and carried that the treasurer be authorized to pay the Board of Censors $25.00 for expenses incurred. Dr. C. A. Ring moved that a committee to consist of Drs. A. L. Benedict, Ira C. Brown and W. C. Callanan be appointed to report at the next annual meeting upon the use of the Metric System in Prescription Writing by the General Practitioner. Carried.

Dr. STORCK presented the following amendment to the by-laws, to be acted upon at the next regular meeting, under the rules:

To amend Sec. 3, Art. VII., of the By-Laws, by adding after the word "both" on the third last line the following:

Or with any practitioner who does not become a member of a legal medical society of the county or state after practising in this county six months, or with any one who has been expelled from any such society.

Any member of the society who is found guilty of violating this section, or of gross unprofessional conduct in violation of the Code of Medical Ethics, after due investigation of the charges by the Board of Censors, and after such specified charges shall have been submitted to the society at any regular or special meeting, and due notice has been served upon the accused by the chairman of the board, to appear before the society in defense — shall be expelled from the society upon a vote of a majority of the members present.

Specific charges in writing, by Dr. C. H. W. Auel, against Dr. D. W. Harrington, for professional misconduct and violation of the Code of Ethics, were presented and read. Upon motion they were referred to the Board of Censors.

In view of the above charges, and for the purpose of having an early consideration of the same, it was moved and carried that when the Society adjourned, it be to meet July 7th, at 8.15 P. M. The Society then adjourned.

